# Effect of the versatile bifunctional chelator AAZTA^5^ on the radiometal labelling properties and the in vitro performance of a gastrin releasing peptide receptor antagonist

**DOI:** 10.1186/s41181-020-00115-8

**Published:** 2020-11-30

**Authors:** Michael Hofstetter, Euy Sung Moon, Fabio D’Angelo, Lucien Geissbühler, Ian Alberts, Ali Afshar-Oromieh, Frank Rösch, Axel Rominger, Eleni Gourni

**Affiliations:** 1 Department of Nuclear Medicine, Inselspital, Bern University Hospital, Freiburgstr. 18, 3010 Bern, Switzerland; 2 Department of Chemistry – TRIGA site, Johannes Gutenberg - University Mainz, Mainz, Germany

**Keywords:** Gastrin releasing peptide receptor (GRPr), AAZTA, Prostate cancer, Imaging, Peptide radionuclide therapy

## Abstract

**Background:**

Gastrin Releasing Peptide receptor (GRPr)-based radioligands have shown great promise for diagnostic imaging of GRPr-positive cancers, such as prostate and breast.

The present study aims at developing and evaluating a versatile GRPr-based probe for both PET/SPECT imaging as well as intraoperative and therapeutic applications. The influence of the versatile chelator AAZTA^5^ on the radiometal labelling properties and the in vitro performance of the generated radiotracers were thoroughly investigated.

The GRPr-based antagonist D-Phe-Gln-Trp-Ala-Val-Gly-His-Sta-Leu-NH_2_ was functionalized with the chelator 6-[Bis (carboxymethyl)amino]-1,4-bis (carboyxmethyl)-6-methyl-1,4-diazepane (AAZTA^5^) through the spacer 4-amino-1-carboxymethyl-piperidine (Pip) to obtain AAZTA^5^-Pip-D-Phe-Gln-Trp-Ala-Val-Gly-His-Sta-Leu-NH_2_ (LF1). LF1 was radiolabelled with gallium-68 (PET), indium-111 (SPECT, intraoperative applications) and lutetium-177 (therapy, SPECT). In vitro evaluation included stability studies, determination of lipophilicity, protein-binding studies, determination of K_d_ and B_max_ as well as internalization studies using the epithelial human prostate cancer cell line PC3. In vitro monotherapy as well as combination therapy studies were further performed to assess its applicability as a theranostic compound.

**Results:**

LF1 was labelled with gallium-68, indium-111 and lutetium-177 within 5 min at room temperature (RT). The apparent molar activities (A_m_) were ranging between 50 and 60 GBq/μmol for the ^68^Ga-labelled LF1, 10–20 GBq/μmol for the ^111^In- and ^177^Lu-labelled LF1. The radiotracers were stable for a period of 4 h post labeling exhibiting a hydrophilic profile with an average of a LogD_octanol/PBS_ of − 3, while the bound activity to the human serum protein was approximately 10%. ^68/nat^Ga-LF1, ^177/nat^Lu-LF1 and ^111/nat^In-LF1 exhibited high affinity for the PC3 cells, with K_d_ values of 16.3 ± 2.4 nM, 10.3 ± 2.73 nM and 5.2 ± 1.9 nM, respectively, and the required concentration of the radiotracers to saturate the receptors (B_max_) was between 0.5 and 0.8 nM which corresponds to approximately 4 × 10^5^ receptors per cell. Low specific internalization rate was found in cell culture, while the total specific cell surface bound uptake always exceeded the internalized activity. In vitro therapy studies showed that inhibition of PC3 cells growth is somewhat more efficient when combination of ^177^Lu-labelled LF1 with rapamycin is applied compared to ^177^Lu-laballed LF1 alone.

**Conclusion:**

Encouraged by these promising in vitro data, preclinical evaluation of the LF1 precursor are planned in tumour models in vivo.

## Background

The need to diagnose, prevent, and treat cancer is greater than ever, since cancer-associated deaths are still a major cause of deaths. Early detection is essential for effective treatment of the disease, hence, many efforts have been made to identify cancer related targets and to further investigate their implication in cancer management (Kumar et al. 2006). The G protein-coupled receptors (GPCR) are of high importance since they have been identified as valuable targets for the development of specific radiotracers for solid tumor imaging in the field of nuclear medicine. The fundamental component for the successful development of GPCR-based radioligands for in vivo tumor imaging is the GPCR overexpression on tumors in combination with their low expression and limited density in the surrounding healthy organs (Reubi 2003), with somatostatin receptor being the first to be defined for in vivo imaging (Gibril et al. 1996). The successful clinical translation of somatostatin receptor targeting was the driving force for the identification of additional GPCR with the potential to be used for peptide receptor targeted diagnostic and therapeutic applications (Reubi 2013). Gastrin Releasing Peptide receptor (GRPr) that is overexpressed on a variety on human tumors, represents another target with high interest in nuclear medicine (Yu et al. 2013).

A high number of GRPr-based radiotracers, both agonists and antagonists, mainly derived from the N-terminal truncated octapeptide bombesin (7–14), have shown promising results (Mansi et al. 2016). GRPr-based radioagonists were the first to be developed. Although extensive preclinical research has been reported, only few radiotracers have been clinically assessed, mainly because of their mitogenic properties and the side effects. The diagnostic accuracy of the ^68^Ga-labelled BZH3 was investigated in patients with gastrointestinal stromal tumors and gliomas (Dimitrakopoulou-Strauss et al. 2007; Strauss et al. 2012). Clinical trials have also been performed with the GRPr agonist AMBA, labelled with gallium-68 and lutetium-177 in patients with various cancers, to assess its applicability as diagnostic as well as therapeutic radiopharmaceutical (Baum et al. 2007; Bodei et al. 2007). Furthermore, the feasibility of RP527 labelled with technetium-99 m was evaluated in patients with prostate and breast cancer (Van de Wiele et al. 2001).

The significant finding of the somatostatin radioantagonists being preferable to radioagonists for in vivo peptide receptor targeting (Ginj et al. 2006) stimulated the shift towards the development of GRPr antagonists too (Cescato et al. 2008). A plethora of radioantagonists have been evaluated preclinically and since 2013 several clinical trials have been performed using GRPr radioantagonists (RM2 (or BAY 86-7548), RM26, NODAGA-MJ9, SB3, NeoBOMB1, TE2AAR06, BAY 86-4367) labelled with gallium-68, fluorine-18, cupper-64, lutetium-177, with RM2 radiolabelled with gallium-68 being the most deliberated one (Kähkönen et al. 2013; Roivainen et al. 2013; Wieser et al. 2014; Sah et al. 2015; Maina et al. 2016; Nock et al. 2017; Gnesin et al. 2018; Minamimoto et al. 2018; Zhang et al. 2018; Fassbender et al. 2019; Touijer et al. 2019). Baratto et al. recently used RM2 radiolabelled with gallium-68 to map its physiological distribution in humans. Pancreas was the organ with the highest uptake, followed by mild to moderate uptake in the gastrointestinal tract while the radiotracer is eliminated through the urinary tract (Baratto et al. 2020). The above radioantagonists were evaluated in patients with newly diagnosed prostate cancer (PC) and at biochemical recurrence of prostate cancer (BCR PC). In summary, the studies outline that the radioantagonists can be safely administrated to patients. They showed high detection rate of primary PC and BCR PC, were able to detect more lesions compared to [18F] fluoroethylcholine and [99mTc]Tc-MDP, apparent performance was improved when compared with the radioagonist ^68^Ga-BBN and exhibited higher detection rates compared to MRI. RM2, RM26 and SB3 labelled with gallium-68 have also been used in few pilot studies to evaluate their detection rate in patients with primary breast cancer (Maina et al. 2016; Stoykow et al. 2016; Zang et al. 2018). These studies suggest that GRPr expression is correlated with estrogen receptors (ER) positive tumors, paving thus the way to expand additional the use of GRPr antagonists as a therapeutic tool.

Peptide receptor radionuclide therapy (PRRT) consists an important treatment option especially in the management of inoperable and metastatic tumors (Kwekkeboom et al. 2008). However, several approaches, such as changes of the radioligand, dosimetry and combination therapy with different agents such as radiosensitisers, have been investigated aiming at improving the response rate and survival. Radiosensitization is considered an outstanding addition in cancer treatment. Inhibition of the phosphoinositide 3-kinase protein kinase/mammalian target of rapamycin (PI3K/Akt/mTOR) pathway, which could be activated by PRRT, causes radiosensitization (Gomez-Pinillos and Ferrari 2012). PI3K/Akt/mTOR regulates several physiological functions such as control of cell metabolism, growth, proliferation and survival. Studies also report that PI3K/Akt/mTOR is activated, in particular in 30%–50% of PC, mainly due to the amplification of gene encoding components, advocating that targeted inhibition of those individual components of the signaling cascade might be a solid strategy for cancer therapy (Gomez-Pinillos and Ferrari 2012). Rapamycin which is a mTOR inhibitor, inhibits tumor growth, angiogenesis, metastasis and causes apoptosis in cancer cell lines as well as in tumor mouse models (Konings et al. 2009). The so far attempts to improve the treatment effect of prostate cancer, when using GRPr-analogues, involve dosimetry studies. The first in human dosimetry study with ^177^Lu-labelled RM2 was recently published. The therapy was well tolerated without side effects. Pancreas was the dose-limiting organ, bone metastases had the highest uptake, followed by lymph nodes and soft tissue lesions (Kurth et al. 2020). These encouraging data pave the way for more intensive efforts, preclinicaly as well as in clinical settings, investigating several treatment options of PC.

**Fig. 1:**
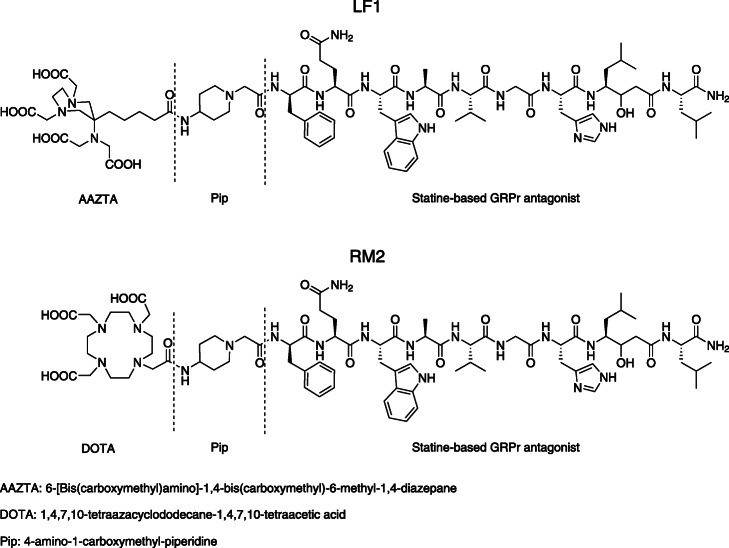
Schematic structures of LF1 and RM2

Herein, the antagonistic statine-based GRPr peptide H-D-Phe-Gln-Trp-Ala-Val-Gly-His-Sta-Leu-NH_2_ was functionalized with the chelator 6-[Bis (carboxymethyl)amino]-1,4-bis (carboyxmethyl)-6-methyl-1,4-diazepane (AAZTA^5^) via the positively charged spacer 4-amino-1-carboxymethyl-piperidine (Pip) to obtain AAZTA^5^-Pip-D-Phe-Gln-Trp-Ala-Val-Gly-His-Sta-Leu-NH_2_ (LF1) (Fig. 1). LF1 was radiolabelled with gallium-68, indium-111 and lutetium-177. One of our goals was to investigate the influence of the versatile bifunctional chelator AAZTA^5^ on the GRPr-based antagonist with regard to its receptor binding affinity and the in vitro performance of the generated radiotracers. Furthermore, the assessment of its potential in serving as theranostic compound for GRPr-positive tumors, in in vitro monotherapy as well as combination therapy studies in the presence of rapamycin, is also reported. The potent GRPr based antagonist RM2 was used as the reference compound (Fig. 1).

## Materials and methods

### Reagents and instrumentation

All reagents and solvents were of the best grade available and were obtained from Sigma-Aldrich, Merck, Fluka, AlfaAesar, VWR, AcrosOrganics and Fisher Scientific and used without further purification. They were provided with a septum. All culture reagents were from Gibco BRL, Life Technologies (Grand Island, NY). [177Lu]Lu^3+^ and [111In]In^3+^ were obtained from Isotope Technologies Garching GmbH (ITG) (Munich, Germany) and b.e.Imaging GmbH (Baden-Baden, Germany), respectively. The GalliaPharm® Ge-68/Ga-68 generator was available from Eckert & Ziegler (Berlin, Germany).

Deuterated solvents for Nuclear Magnetic Resonance (NMR) spectra were commercially available by Deutero. Thin layer chromatography plates from Merck, silica gel 60 F254 coated aluminium plates, were used for the analysis. Silica gel 60 (core size 0.063 / 0.200 mm) from Macherey-Nagel was used for purification by column chromatography.

The purification of the peptide was performed by semipreparative Reverse-Phase High Performance Liquid Chromatography (RP-HPLC) on a 120–5 C18 Nucleosil column (250 × 21 mm) applying a linear gradient of 15–90% solvent B in 25 min at a flow rate of 12 mL / min (solvent A, 0.1% trifluoroacetic acid (TFA) / water (H_2_O); solvent B, 0.1%TFA / Acetonitrile (ACN).

The quality control of the peptides as well as the radiolabelled compounds was performed by analytical RP-HPLC from Knauer advanced scientific instrument equipped with a Knauer Smartline Manager 5000, a Smartline Pump 1000 and a Smartline UV Detector 2600. The RP-HPLC runs were performed on an analytical 120–5 C18 Nucleosil column (250 × 4.5 mm) applying a linear gradient of 15–90% solvent B in 25 min at a flow rate of 1 mL / min. (solvent A, 0.1% TFA / H_2_O; solvent B, 0.1% TFA / ACN). Ultraviolet detection was performed using a Knauer detector at 240 nm. For radioactivity measurement, a Na (TI) well-type scintillation Gina star was used. The radiotracer solutions were prepared by dilution with 0.9% NaCl.

The human prostate adenocarcinoma cell line PC3 was obtained from CLS Cell Lines Service GmbH, (Eppelheim, Germany). Cell culture media Dulbecco’s Modified Eagle Medium (DMEM) with GlutaMax-I Supplement, F-12 Nutrient Mixture with GlutaMax-I Supplement, Dubecco’s Phosphate Buffered Saline (DPBS), Fetal Bovine Serum (FBS), Trypsin-EDTA and antibiotic solution Penicillin-Streptomycin were from Gibco BRL, Life Technologies (Grand Island, NY) and purchased from ThermoFisher (Switzerland).

Electrospray Ionization - Mass Spectrometry (ESI-MS) mass spectra were acquired on a Bruker Daltonics Esquire 3000 plus device.

The ^1^H-, ^13^C-NMR measurements were performed on a Bruker Avance III HD 400 (400 MHz) or Avance III 600 (600 MHz). LC / MS spectra were measured on an Agilent Technologies 1220 Infinity LC system coupled to an Agilent Technologies 6130B Single Quadrupole Liquidchromatography - Mass spectrometry (LC / MS) system.

Thin Layer Chromatography (TLC) scans were acquired on an Elysia Raytest TLC scanner using the Gina Star TLC software.

Quantitative γ-counting was performed with a Cobra II Gamma Counter from Packard Instrument (USA).

All experiments were carried out twice in triplicates.

### Synthesis of the prochelator AAZTA^5^-(^t^Bu)_4_

**Fig. 2:**
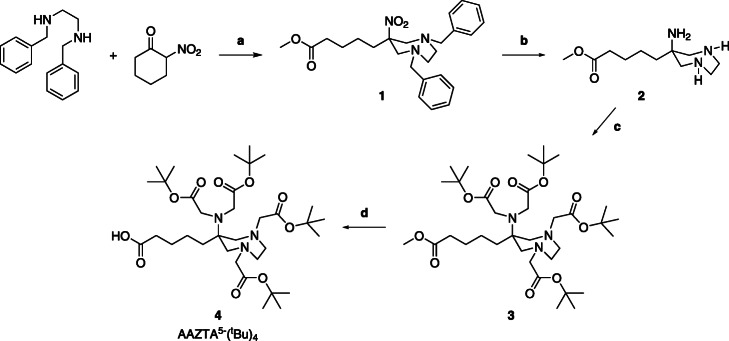
Synthetic scheme of AAZTA^5^-(^t^Bu)_4_: **a** paraformaldehyde, MeOH, Amberlyst A21; **b** Pd (OH)_2_/C, CH_3_COOH, EtOH, K_2_CO_3_; **c** tert-butylbromacetate, MeCN, K_2_CO_3_, KI; **d** 1 M LiOH, 1,4-dioxane/H_2_O (2:1)

The precursor AAZTA^5^-(^t^Bu)_4_ was successfully synthesized over 4-steps following the protocol described by Sinnes et al. (2019b). Briefly, the synthesis steps are described below and shown in Fig. 2:

- *1,4-Dibenzyl-6-methylpentanoate-6-nitroperhydro-1,4-diazepane* (**1**)
  - 2-nitrocyclohexanone (2.00 g; 13.9 mmol) and Amberlyst A21 (1.05 g) were mixed with dry methanol (MeOH) (35 mL). The solution was heated to 60 °C and stirred under reflux for 1 h. *N,N′*-dibenzylethylene-diamine (3.36 g; 13.9 mmol) and paraformaldehyde (1.67 g; 55.5 mmol) were added to the solution. The suspension was heated to 80 °C and stirred overnight. After completion of the reaction, the suspension was filtered and the filtrate concentrated under vacuum. After column chromatographic purification (Cyclohexane (CH) / Ethyl Acetate (EA); 9:1; R_f_ = 0.27) a yellow oil was obtained as product **1** (5.20 g; 11.8 mmol; 85%).
- *1,4-Di (tert-butylacetate)-6-methylpentanoate-6-amino-di (tert-butylacetate)-perhydro-1,4-diazepine* (**3**)
  - **1** (1.05 g, 2.39 mmol) was added to a solution containing Pd (OH)_2_ / C (0.62 g, 10 wt%) and abs. Ethanol (EtOH) (20 mL). Acetic acid (411 μL; 7.18 mmol) was added, the solution was saturated with hydrogen and stirred overnight at room temperature. After completion of the reaction, Pd (OH)_2_ / C was filtered over Celite and the filtrate was concentrated under vacuum. Crude product **2**, a white-yellowish solid, was used for further reaction without further purification.
  - **2** (1.05 g; 2.39 mmol) and K_2_CO_3_ (1.32 g; 9.57 mmol) were dissolved in dry ACN (30 mL). *tert*-butyl bromoacetate (1.41 mL; 9.57 mmol) and potassium iodide (0.80 g) were added and stirred overnight at 40 °C. The solution was concentrated under vacuum and purified by column chromatography (CH / EA, 7:1; R_f_ = 0.15). Compound **3** was obtained as yellow oil (0.89 g; 1.30 mmol; 54%).
- *1,4-Di (tert-butylacetate)-6-pentanoicacid-6-(amino-di (tert-butylacetate))-perhydro-1,4-diazepine* (**4**)
  - **3** (293 mg, 0.43 mmol) was dissolved in 1,4-dioxane / H_2_O (2:1; 6 mL). 1 M LiOH solution (641 μL; 0.64 mmol) was added and stirred overnight at RT. After completion of the reaction, the solution was concentrated, the residue concentrated with 1 M NaHCO_3_ solution and extracted several times with chloroform. The organic phase was extracted with H_2_O, dried with Mg_2_SO_4_ and concentrated under vacuum. Compound **4** was obtained a yellowish oil (224 mg; 0.33 mmol; 76%).

### Synthesis of the chelator-peptide conjugate

The peptide-chelator conjugate was synthesized manually using standard fluorenylmethoxycarbonyl protecting group (Fmoc) chemistry and Rink amide 4-methylbenzhydrylamine resin. The spacer (Pip) and the prochelator (AAZTA^5^-(^*t*^Bu)_4_) were consecutively coupled to the peptide using 1-[Bis (dimethylamino)methylene]-1H-1,2,3-triazolo[4,5-b] pyridinium 3-oxid hexafluorophosphate (HATU) as an activating agent. The cleavage of the peptide and the simultaneous deprotection of the side chain-protecting groups was performed using TFA / triisopropylsilane (TIPS) / H_2_O (95 / 2.5 / 2.5). The crude conjugate was further purified by semipreparative RP-HPLC as described in the “Reagents and Instrumentation” section.

### Radiochemistry

#### ^68^Ga-labelled LF1

The ^68^Ga-labelled radiotracer was prepared within 5 min, using the Modular-Lab PharmTracer module by Eckert & Ziegler (Berlin, Germany). The radiolabeling performance of LF1 was assessed at pH 4.0 (0.2 M sodium acetate buffer), at RT using a conjugate amount of 5–20 μg (approximately 3–12 nmol). Briefly, the Ge-68/Ga-68 generator was eluted with 5 mL HCl 0.1 N and the eluate (~ 400 MBq) was loaded onto a cation exchange column (Strata-XC, Phenomenex). Gallium-68 was eluted with 700 μL of a mixture of 5.5 M NaCl / 0.1 M HCl directly in a vial containing 400 μL of 1.8 M sodium acetate buffer, 2 mL H_2_O, 200 μL of EtOH, 5 mg ascorbic acid / 20 μL H_2_O and the tested amount of the conjugate, followed by SepPak C-18 purification to remove uncomplexed radiometal.

#### ^177^Lu- and ^111^In-labelled LF1 and ^177^Lu-labelled RM2

The ^177^Lu- and ^111^In-labelled radiotracers were prepared by dissolving 5–10 μg (approximately 3–6 nmol) of LF1 in 250 μL ammonium acetate buffer (0.5 M, pH 5.4), followed by incubation with [^177^Lu]Lu^3+^ (30–100 MBq) or [^111^In]In^3+^ (approximately 35–40 MBq) for 10 min at RT and were used without any further purification step.

After the labelling with gallium-68, lutetium-177 or indium-111 and the quality control of the generated radiotracers, one equivalent of either ^nat^Ga (NO_3_)_3_ or ^nat^LuCl_3_ x 6H_2_O or ^nat^InCl_3_ x 2H_2_O were added to the relevant radiolabelling solutions. The final solutions were incubated at RT for 10 min to obtain structurally characterized homogeneous ligands which were used for the saturation binding studies. The homogeneity was determined by HPLC, showing one peak.

### Quality control of the radiotracers / stability

Chemical and radiochemical purity of the tested solutions were determined using an analytical Nucleosil 100–5 C18 column applying the conditions described in the “Reagents and Instrumentation” section.

The presence of free gallium-68 and ^68^Ga-labelled colloid in the ^68^Ga-labelled LF1 preparation was quantified by radio thin layer chromatography (Radio-TLC) using Silica gel 60-plates and two different mobile phase eluents: a) 0.1 M Na-citrate; b) MeOH / 1 M ammonium acetate (1 / 1, v / v). Using the first Radio-TLC eluent, the radiopeptide product and ^68^Ga-labelled colloid remain immobilized at the starting point, whereas free gallium-68 move with the mobile phase. When the second eluent is used, only the labelled peptide moves with the mobile phase / solvent front.

The stability of the newly prepared radiotracers was tested via RP-HPLC for a period of 4 h post labeling.

### Lipophilicity

The lipophilicity (LogD_octanol/PBS_, pH 7.4) was estimated by the “shake-flask” method: The labelled conjugates (100 pmol; 1.1 MBq, 1.2 MBq and 0.5 MBq for ^68^Ga-, ^177^Lu- and ^111^In-labelled LF1 respectively) were added to a solution of 1-octanol (500 μL) and of PBS (500 μL, pH 7.4). The mixture was vortexed for 1 h to reach the equilibrium and then centrifuged (3000 rpm) for 10 min. From each phase, an aliquot (50 to 100 μL) was pipetted out and measured in a gamma-counter. Each measurement was repeated five times. Care was taken to avoid cross-contamination between the phases. The partition coefficient was calculated as the average log ratio of the radioactivity in the organic fraction and the PBS fraction.

### Protein binding studies in human plasma

^68^Ga-, ^177^Lu- and ^111^In-labelled LF1 (100 pmol; 1.1 MBq, 1.2 MBq and 0.5 MBq, respectively) were incubated with human plasma (0.5 mL) at 37 °C for 30 min and 60 min respectively. When the incubating period was completed, proteins were precipitated with a solution of 1 mL of MeOH / ACN (1:1). Centrifugation (10 min, 9660 g) for the separation of proteins was then performed. After careful separation of the two phases, the respective activities were measured in a gamma-counter, followed by determination of the percentage of each radiotracer which does not bind to the plasma proteins.

### Cell culture

The human prostate epithelial adenocarcinoma cell line PC3, which is known to overexpress GRPr, was cultured at 37 °C and 5% CO_2_ in Dulbecco’s Modified Eagle Medium (DMEM) with GlutaMAX-I supplement and F-12 Nutrient Mix with GlutaMAX-I supplement in a ratio of 1:1. The medium was supplemented with 10% fetal bovine serum (FBS), penicillin (100 U / mL) and streptomycin (100 μg / mL).

### Saturation binding studies

For receptor saturation analysis, PC3 cells were seeded at a density of 0.8–1 million cells per well in 6-well plates and incubated overnight with medium (DMEM: F12 (1:1) containing 1% FBS, 100 U/mL penicillin and 100 μg/mL streptomycin). The next day, the medium was removed, the cells washed and incubated for 1 h at 37 °C with fresh medium. Afterwards, the plates were placed on ice for 30 min followed by incubation with increasing concentrations of either ^68/nat^Ga-LF1 or ^177/nat^Lu-LF1 or ^111/nat^In-LF1 or ^177/nat^Lu-RM2 (1–100 nM) in phosphate-buffered saline binding buffer pH 7.4. After the addition of the radioligands, the cells were incubated for 120 min at 4 °C. Non-specific binding was determined in the presence of Tyr^4^-Bombesin at a final concentration of 1 μM. Then the cells were washed twice with ice-cold PBS, followed by solubilization with 1 N NaOH and the cell-associated radioactivity was measured using a gamma-counter. Specific binding was plotted against the total molar concentration of the added radiotracer. The K_d_ values and the concentration of the radiotracer required to saturate the receptors (B_max_) were determined by nonlinear regression using GraphPad (Prism 8 Graph Pad Software, San Diego, CA). For all the cell studies the values are normalized for 1 × 10^6^ cells per well and all data are from two independent experiments with triplicates in each experiment.

### Internalization studies

For internalization experiments, PC3 cells were seeded into 6-well plates and treated as described above. Approximately 0.25 pmol of the respective radiopeptides were added to the medium and the cells were incubated (in triplicates) for 0.5, 1, 2, 4 and 6 h at 37 °C, 5% CO_2_ for ^177^Lu- and ^111^In-labelled LF1 and for 15, 30, 60, 90, 120, 180 and 240 min at 37 °C, 5% CO_2_ for ^68^Ga-labelled LF1. To determine nonspecific membrane binding and internalization, excess of Tyr^4^-Bombesin (final concentration 1 μΜ) was added to selected wells. At each time point, the internalization was stopped by removing the medium and washing the cells twice with ice-cold PBS. To remove the receptor-bound radioligand, an acid wash was carried out twice with a 0.1 M glycine buffer pH 2.8 for 5 min on ice. Finally, cells were solubilized with 1 N NaOH. The radioactivity of the culture medium, the receptor-bound, and the internalized fractions were measured in a γ-counter.

### In vitro therapy studies with ^177^Lu-labelled LF1 in the presence or without rapamycin

For the in vitro therapy studies, PC3 cells were seeded at a density of 50,000–100,000 cells (depending on the investigating time point: 100000, 75,000 and 50,000 cells for 24, 48, 72 h respectively) in 12-well plates and incubated overnight with cultivation medium at 37 °C and 5% CO_2_. The next day, the medium was removed, the cells washed, and culture medium containing either rapamycin (10 nM, dissolved in dimethyl sulfoxide (DMSO)) or DMSO (control, the final concentration of DMSO in the culture medium was 0.5%) was added. The plates were incubated at 37 °C and 5% CO_2_ for a period of 6 h. Afterwards, the medium containing rapamycin or DMSO was removed and fresh medium was added in each well. Furthermore, 1.85 MBq of ^177^Lu-labelled LF1 was added to preselected wells. The monotherapy (either with rapamycin or ^177^Lu-labelled LF1 alone) and combination therapy study (^177^Lu-labelled LF1 in presence of rapamycin) were performed in parallel with untreated cells which served as reference. The viability of the cells was accessed by the trypan blue exclusion assay after incubation at 37 °C and 5% CO_2_ for 24, 48 and 72 h. The experiment was performed twice in triplicate.

### Statistical analysis

All data are expressed as the mean of values ± standard deviation (mean ± SD). Prism 8 Software (GraphPad Software) was used to determine statistical significance at the 95% confidence level, with a *P* value of less than 0.05 being considered significant.

## Results

### Synthesis of the prochelator AAZTA^5^-(^t^Bu)_4_

The synthesis route of AAZTA^5^-(^t^Bu)_4_ is shown in Fig. 2. First, ring opening of 2-nitrocyclohexanone was carried out in situ*.* A diazepane ring was formed by reaction with *N,N′*-dibenzyldiamine via a double nitro-Mannich reaction, **1**. Hydrogenolysis using Pd (OH)_2_ / C and H_2_ removed the benzyl protecting groups at the endocyclic amines and simultaneously reduced the nitro group to an amine, **2**. The unstable product was directly reacted with *tert*-butyl bromoacetate without further purification to form tetra alkylated compound, **3**. Deprotection of the methyl ester protecting groups were carried out by using 1 M lithium hydroxide solution and 1,4-dioxane / H_2_O (ratio 2:1) to receive the bifunctional chelator AAZTA^5^-(^*t*^Bu)_4_, **4**.

The analytic data of the intermediate products **1** and **3** as well as the prochelator **4** are presented below:

- Product **1**:
  - MS (ESI^+^): m/z (%): 440.3 (M + H^+^); calculated for C_25_H_33_N_3_O_4_: 439.25
  - ^1^H-NMR (CDCl_3_, 400 MHz, δ [ppm]): 7.29 (m, 10 H); 3.66 (s, 3 H); 3.67 (dd, J = 13.5 Hz, 4 H); 3.25 (dd, J = 14.0 Hz, 4 H); 2.63 (m, 4 H); 2.12 (m, 2 H); 1.59 (m, 2 H); 1.32 (m, 2 H); 0.78 (m, 2 H). ^13^C-NMR (CDCl_3_, 100 MHz, δ [ppm]): 173.6 (s); 139.1 (s); 129.1 (s); 128.3 (s); 127.3 (s); 94.8 (s); 64.9 (s); 61.8 (s); 58.9 (s); 51.5 (s); 36.5 (s); 33.6 (s); 24.6 (s); 22.6 (s).
- Product **3**:
  - MS (ESI^+^): m/z (%): 686.5 (M + H^+^); 708.4 (M + Na^+^); calculated for C_35_H_63_N_3_O_10_: 685.45
  - ^1^H-NMR (CDCl_3_, 400 MHz, δ [ppm]): 3.65 (s, 4 H); 3.61 (s, 4 H); 3.22 (s, 3 H); 2.99 (d, J = 14.1 Hz, 2 H); 2.85–2.65 (m, 4 H); 2.63 (d, J = 14.1 Hz, 2 H); 2.31 (t, J = 7.4 Hz, 2 H); 1.62–1.52 (m, 4 H); 1.44 (s, 18 H); 1.43 (s, 18 H); 1.25 (m, 2 H). ^13^C-NMR (CDCl_3_, 100 MHz, δ [ppm]): 174.4 (s); 172.9 (s); 170.9 (s); 80.9 (s); 80.4 (s); 65.3 (s); 63.2 (s); 62.6 (s); 59.4 (s); 52.1 (s); 51.6 (s); 37.3 (s); 34.3 (s); 28.3 (s); 28.3 (s); 25.9 (s); 21.8 (s).
- Product **4** (AAZTA^5^-(^t^Bu)_4_):
  - MS (ESI^+^): m/z (%): 672.4 (M + H^+^); 694.5 (M + Na^+^); calculated for C_34_H_61_N_3_O_10_: 671.44
  - ^1^H-NMR (CDCl_3_, 400 MHz, δ [ppm]): 3.60 (s, 4 H); 3.23 (s, 4 H); 3.00–2.97 (d, J = 14.1 Hz, 2 H); 2.88–2.60 (m, 6 H); 2.36–2.32 (t, J = 7.9 Hz, 2 H); 1.64–1.52 (m, 4 H); 1.43 (s, 18 H); 1.42 (s, 18 H); 1.24 (m, 2 H). ^13^C-NMR (CDCl_3_, 100 MHz, δ [ppm]): 178.9 (s); 172.9 (s); 170.9 (s); 81.0 (s); 80.5 (s); 65.1 (s); 63.1 (s); 59.4 (s); 52.2 (s); 34.2 (s); 29.8 (s); 28.3 (s); 28.2 (s); 25.6 (s); 22.8 (s); 21.9.

### Synthesis of the chelator-peptide conjugate

The synthesis yields of the peptide-chelator conjugate ranged from 30 to 40%. The purity and identity of the peptids was > 95% as determined by HPLC and mass spectroscopy. The analytical data are depicted in Table 1.

**Table 1:** Analytical data of LF1 and RM2

Compound	Elemental composition	Purity	Calculated mass	MS (ESI)	Rt (min)
**LF1**	C_80_H_116_N_18_O_22_	> 95%	1682.89 m/z [M + H^+^]	1623.89 m/z	13.8
**RM2**	C_78_H_118_N_20_O_19_	> 95%	1638.89 m/z [M^+**·**^]	1638.89 m/z	12.8

A reception control (HPLC and mass spectroscopy) of the compound RM2 (GMP grade) was also performed at our department and the analytical data are reported in Table 1.

### Radiochemistry / quality control / stability

#### ^68^Ga-labelled LF1

LF1 (3, 6 and 12 nmol) was labelled with gallium-68 in about 90% radiochemical purity as determined by analytical HPLC, using a UV absorption at λ = 240 nm. The formation of one additional radioactive species (about 10%) was also observed. The retention time of the main peak was 12.8 min while the retention time of the second radioactive species was 12.1 min (Figure 1S). TLC excluded the creation of ^68^Ga-labelled colloids (Figure 1S). The maximum achieved apparent molar activity (A_m_) was approximately 60 GBq / μmol.

#### ^177^Lu- and ^111^In-labelled LF1

LF1 was labelled with lutetium-177 and indium-111 at RT in 98% radiochemical purity as determined by analytical HPLC, using a UV absorption at λ = 240 nm. Both analytical methods, HLPC and TLC verified the presence of one radioactive species (the corresponding radiolabelled peptide in each case) while the percentage of the free metal was < 2%. The retention times of ^177^Lu- and ^111^In-labelled LF1 were 12.7 and 14.5 min respectively (Figure 1S). The apparent molar activities (A_m_) were ranging between 10 and 20 GBq / μmol for ^177^Lu-labelled LF1 and approximately 10 GBq / μmol for the ^111^In-labelled radioligand.

The stability of all the radiotracers over time was determined with RP-HPLC and neither radiolysis or decomposition was observed for a period of 4 h post labeling.

### Lipophilicity / protein binding studies in human plasma

With a LogD_octanol/PBS_ of − 3.2 ± 0.04, − 2.9 ± 0.04 and − 2.8 ± 0.08 for the ^68^Ga-, ^177^Lu- and ^111^In-labelled LF1, respectively, all the radiotracers show a hydrophilic profile. (Lipophilicity = the logarithm of the partition coefficient D, where D is the ratio of the distribution of a compound in 2 solvents, here octanol and PBS).

To estimate the bioavailability of the three radiotracers in circulation, the extent of human plasma protein binding was determined. Approximately 10% of the incubating gallium-68, lutetium-177and indium-111 activity was found to be bound to plasma proteins at the tested time points.

### Saturation binding studies

**Fig. 3:**
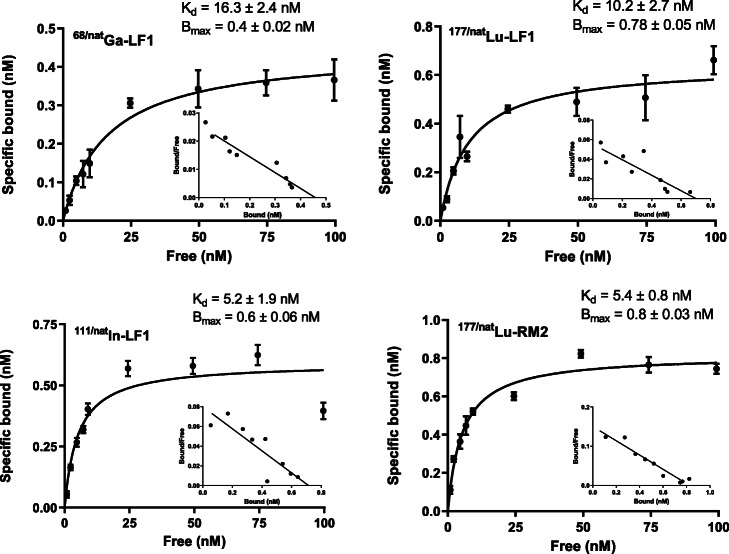
Saturation binding study on intact PC3 cells, using increasing concentrations of ^68/nat^Ga-LF1, ^177/nat^Lu-LF1, ^111/nat^LF-LF1 and ^177/nat^Lu-RM2, ranging from 1 to 100 nM. Dissociation constant (K_d_) and maximum number of binding sites (B_max_) were calculated from nonlinear regression analysis using GraphPad Prism

Saturation binding studies were performed at 4 °C, in order to allow binding of the radioconjugates to the receptor but to avoid endocytosis. All the radiotracers exhibited affinity for the GRPr positive PC3 cells, with K_d_ values of 16.3 ± 2.4 nM for ^68/nat^Ga-LF1, 10.2 ± 2.7 nM for ^177/nat^Lu-LF1 and 5.2 ± 1.9 nM for ^111/nat^In-LF1 (Fig. 3). The B_max_ values were also at the same level for the three radiotracers (0.4 ± 0.02 nM for ^68/nat^Ga-LF1, 0.8 ± 0.05 nM for ^177/nat^Lu-LF1 and 0.6 ± 0.06 nM for ^111/nat^In-LF1) which correspond to approximately 4 × 10^5^ receptors per cell.

The side-by-side comparison with the high affinity GRPr antagonist RM2 revealed that the affinities of the new tested radiotracers were at the same range with ^177/nat^Lu-RM2 (K_d_: 5.4 ± 0.8 nM and B_max_: 0.8 ± 0.03 nM).

### Internalization studies

**Fig. 4:**
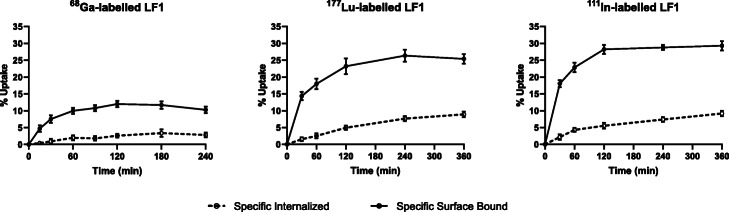
Internalization rate and specific surface bound uptake after the incubation of PC3 cells with ^68^Ga-, ^177^Lu-, ^111^In-labelled LF1 within 4 or 6 h at 37 °C. Receptor-specific internalization and specific surface bound activity expressed as percentage of the applied radioactivity. Nonspecific binding was determined in the presence of 1 μM Tyr^4^-BN

^68^Ga-, ^177^Lu- and ^111^In-labelled LF1 were found to be well associated with the PC3 cells within the incubation time frame (Fig. 4). Continued exposure of the cells to the radioactive ligands resulted in a gradual increase of the total cell associated uptake from 15 min to 4 h for ^68^Ga-labelled LF1 and from 30 min to 6 h for ^177^Lu- and ^111^In-labelled LF1. ^111^In-labelled LF1 exhibited the higher total cell associated uptake (38.3 ± 2.5%) (Figure 2S) followed by ^177^Lu-labelled LF1 (34.5 ± 2.3%) at 6 h (*P* = 0.0035) (Figure 2S). ^68^Ga-labelled LF1 revealed the lowest cell associated uptake (13.1 ± 0.24%) (Figure 2S). At 6 h, the amount of specifically internalized activity was 9.2 ± 0.5% for ^111^In-labelled LF1 and 8.9 ± 0.8% for ^177^Lu-labelled LF1 (*P* = 0.0054), while ^68^Ga-labelled LF1 exhibited 2.8 ± 0.5% internalized activity at 4 h. Blocking experiments performed with excess of Tyr^4^-BN, showed negligible nonspecific binding on the cell surface, while less than 0.3% of total added radioactivity was found to be internalized (data not shown) demonstrating the high specificity of the GRPr-conjugates towards PC3 cells.

### In vitro therapy studies with ^177^Lu-labelled LF1 in the presence or without rapamycin

**Fig. 5:**
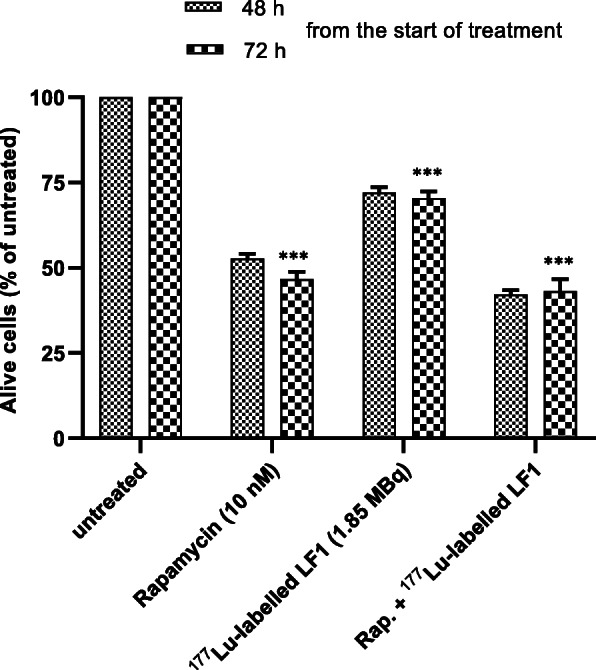
In vitro therapy assessing the effect of monotherapy (rapamycin or ^177^Lu-labelled LF1) and combination therapy (rapamycin and ^177^Lu-laballed LF1) on PC3 cells

An in vitro therapy study was performed aiming at evaluating the effect of combination therapy on PC3 cells (Fig. 5). The viability of PC3 cells was verified 48 and 72 h after treatment. The chosen dose of rapamycin for the in vitro therapy studies was 10 nM, since it has been previously shown that rapamycin at this concentration has the greatest cytostatic effect on PC3 cells (Dumont et al. 2013). The applied dose of ^177^Lu-labelled LF1 was 1.85 MBq. Combination therapy was found to be slightly more effective compared to rapamycin or ^177^Lu-labelled LF1 alone (cell viability: approx. 40%, 48 and 72 h after the treatment). The treatment only with rapamycin showed a higher impact compared to ^177^Lu-labelled LF1 on PC3 cells (cell viability: approx. 50% for rapamycin and 70% ^177^Lu-labelled LF1 72 h).

## Discussion

Prostate cancer, a complex and biologically heterogeneous disease, is the most common type of cancer found in men, which often begins without symptoms. It is on a spectrum varying from asymptomatic and slow-growing to a more aggressive and rapidly progressive systemic malignancy (Kelloff et al. 2009). The survival rate could, therefore, be significantly improved when early detection and personalized treatment take place. Photon emission computed tomography (SPECT) and positron emission tomography (PET) have gained a lot of attention in the management of prostate cancer due to their high accuracy rate. The overexpression of peptide receptors on the surface of prostate cancer cells played a pivotal role in this development and consequently generated interest in the investigation of radiolabelled peptide-based probes for radio-theranostic applications. Prostate Specific Membrane Antigen (PSMA) and GRPr are the two receptors, which are overexpressed on the surface of prostate cancer. Many efforts have been made towards the development of PSMA- and GRPr-based imaging agents with a particular focus on the low molecular weight PSMA inhibitors and the GRPr-based antagonists. PSMA theranostics have proven great success in prostate cancer management in the recent years (Eder et al. 2012; Afshar-Oromieh et al. 2017). However, due to low PSMA expression not all patients suffering from prostate cancer can benefit from the advantages of PSMA targeting. Therefore, there is an urgent need for these patients for both characterization, planning, monitoring of the cancerous disease and treatment itself. Furthermore, because of the noticeable heterogeneity of prostate cancer at the time of diagnosis (Schrecengost and Knudsen 2013), and even more during the recurrence (Scher et al. 2013), it might be possible that the combination of imaging probes would provide clinical advantages with respect to the imaging of various types and stages of prostate cancer. GRPr and PSMA expression were compared on primary prostate cancer samples (Schollhammer et al. 2019; Touijer et al. 2019) as well as patients (Baratto et al. 2020) providing evidence that the biodstribution for the two radioligands were distinct. The so far preliminary data shows that the low metastatic risk PC patients could benefit from GRPr targeting while in high-risk patients the PSMA-based radiotracers could serve as a valuable tool (Minamimoto et al. 2016).

Ongoing research efforts are currently directed to the development of both, PSMA- and GRPr-based radiotracers with a particular focus of bridging radiopharmaceuticals within a theranostic framework. The selection of an appropriate chelator, which bears excellent coordination ability for diagnostic and therapeutic radionuclides, consists the key step of this approach. The most frequently used chelator for radiopharmaceuticals in clinical routine applications, DOTA (1,4,7,10-tetraazacyclododecane-1,4,7,10-tetraacetic acid), has shown great ability for binding a variety of radiometals such as gallium-68, indium-111, yttrium-90, cupper-64, lutetium-177. However, its applicability presents some limitations since efficient radiolabeling yields are achieved between 80 and 100 °C and the optimum labeling pH is 3 to 5 (Price and Orvig 2014). NOTA (1,4,7-triazacyclononane-1,4,7-triacetic acid) and its derivative NODAGA (1,4,7-triazacyclononane, 1-glutaric acid-4,7 acetic acid), enable sufficient labeling yields in lower temperatures compared to DOTA but lower specific activities compared to the DOTA- conjugates are reported. The biggest disadvantage of NOTA and NODAGA is that their cavity is not suitable for the incorporation of therapeutic radionuclides such as lutetium-177 (Eisenwiener et al. 2002). The acyclic chelating agent, HBED-CC (*N,N′*-bis-[2-hydroxy-5-(carboxyethyl)benzyl]ethylenediamine-*N,N′*-diacetic acid) which has been used for the functionalization of PSMA-11, allows efficient radiolabelling with gallium-68 even at ambient temperature, but it forms multiple radiolabelled species (Eder et al. 2014). Therefore, a bifunctional chelator which could surmount the above obstacles would be the optimum choice for the development of a new class of radiotracers. AAZTA^5^, a versatile chelator could be considered as a potential candidate for this purpose. While AAZTA was initially developed for the chelation of Gd (III) as an MRI agent (Aime et al. 2004), it was shown that it exhibits high coordination capability for a variety of metal ions such as gallium-68, indium-111, lutetium-177, scandium-44 (Eisenwiener et al. 2002; Waldron et al. 2013; Pfister et al. 2015; Tsionou et al. 2017; Sinnes et al. 2019a). The mesocyclic structure of AAZTA^5^ facilitates efficient labeling with high molar activities with a variety of radionuclides at RT and even at near neutral pH, optimal conditions for small proteins. Throughout this study, our main goal was to investigate whether and how N-terminal modulations, via the coupling with AAZTA^5^, may influence the affinity of the derived GRPr-based radioligand towards GRPr. A particular focus on additional in vitro studies would allow us to assess the suitability of these radiotracers for further evaluation. Attention was also given on the radiometal labelling properties of LF1 after its labeling with gallium-68 (PET), lutetium-177 (therapy, SPECT) and indium-111 (SPECT, intraoperative applications).

Radiolabelling of LF1 with lutetium-177 and indium-111 supports its applicability for sufficient labeling under mild conditions, even at nearly neutral pH. The high stability in combination with the hydrophilic character may have resulted in the low binding to the proteins of human serum (around 10%) as shown by our in vitro studies.

When LF1 was labelled with gallium-68 two radiolabelled species were observed by radio-HPLC while Waldron et al. reported the presence of three radioactive species when AAZTA was labelled with gallium-68 under the same conditions (Waldron et al. 2013). Since the difference of the retention time between the two observed species was less than 1 min we cannot fully exclude the formation of one additional species. Modification at the iminodiacetate moiety of AAZTA where one of the acetate groups could be replaced by a methyl group may prevent the formation of side products.

The observed differences of ^68^Ga- compared to ^177^Lu- and ^111^In-labelled LF1 with regard to their radiochemical purity prompted us to further investigate if their GRPr binding affinity is influenced and to what extent. Additionally, a side-by-side comparison of their binding affinities along with the binding affinity of ^177/nat^Lu-RM2, which served as a reference peptide, was also performed. ^111/nat^In-LF1 (K_d_: 5.16 ± 1.94 nM) was the most affine exhibiting a K_d_ value similar to ^177/nat^Lu-RM2 (K_d_: 5.42 ± 0.84 nM) followed by ^177/nat^Lu-LF1 (K_d_: 10.25 ± 2.73 nM). ^68/nat^Ga-LF1 (K_d_: 16.27 ± 2.45 nM) was still affine towards GRPr, however, the tracer with the lowest affinity almost by a factor of 3 compared to ^111/nat^In-LF1. Since for AAZTA, the N_2_O_4_ coordination may also occur in a competitive way with the favorable N_3_O_3_ coordination, this might be the reason of the decreased affinity of ^68/nat^Ga-LF1. These findings were further supported by the in vitro internalization studies, which demonstrated high and selective binding of ^68^Ga-, ^177^Lu- and ^111^In-labelled LF1 to GRPr overexpressing cells. ^177^Lu- and ^111^In-labelled LF1 exhibited a comparable profile in terms of total cell associated uptake and percentage of specific internalized fraction while the corresponding values of ^68^Ga-labelled LF1 were considerably lower. The surface associated activity exceeded the amount of internalized activity at all-time points for the three tested radiotracers. In previous studies (Mansi et al. 2011), it was shown that RM2 at a concentration of 10 μM does not cause any effect on calcium mobilization after its incubation with PC3 cells, while an immunofluorescence assay revealed no receptor internalization of the DOTA-Pip-conjugate RM2. The previously confirmed antagonistic potency of RM2 in combination with our findings led us to the assumption that the introduction of AAZTA^5^ did not change the antagonist features of the statine-based motif and that LF1 retains its GRPr-antagonistic profile.

These data may eliminate the applicability of ^68^Ga-labelled LF1 as a PET radiotracer. However, AAZTA may be a suitable chelator for other PET radionuclides, such as scandium-44 (Nagy et al. 2017) and cupper-64 (Greifenstein et al. 2020) and the potential of LF1 to serve as a PET tracer should be further evaluated. Furthermore, scandium-44 and cupper-64 due to their longer half-life (3.97 and 12.7 h, respectively) compared to gallium-68 (67.7 min) allow late imaging. This is of paramount importance especially in case of GRPr expression imaging, since preclinical (Gourni et al. 2014) as well as clinical data (Wieser et al. 2014) have shown that late imaging may be favorable due to the efficient background clearance.

In a next step, the assessment of the potential of LF1 in serving as theranostic compound for GRPr-positive tumors was investigated. An in vitro therapy study was executed with the aim to evaluate the effect of combination therapy on PC3 cells (Edlind and Hsieh 2014) after their treatment with rapamycin, a FDA approved specific inhibitor of mTOR. Our preliminary in vitro data showed that combination therapy was slightly more effective compared to monotherapy with ^177^Lu-labelled LF1. These in vitro data is only an early evaluation of whether rapamycin before ^177^Lu-labelled LF1 treatment would have an additive effect; extensive in vivo studies needs to be performed to safely suggest if the mTOR kinase inhibition may contribute to the sensitization of the malignant prostate cancer cells to radiation.

In vivo studies are planned to be performed in order verify the preliminary in vitro data and further define the ideal therapeutic doses and therapy regimens.

## Conclusion

On the basis of the current results, LF1 labelled lutetium-177 and indium-111 appears to have a considerable potential to serve as a versatile probe suitable for SPECT, therapy and intraoperative applications. The ease of LF1 synthesis, the efficient radiolabeling at RT with a variety of radiometals, the stable encapsulation of lutetium-177 and indium-111 by AAZTA^5^ and their favorable in vitro performance renders them suitable candidates for further extensive in vivo studies.

In vivo studies are planned to be performed which together with the already in vitro data may provide a solid evidence for their clinical translation.

## Supplementary Information

**Additional file 1: Figure 1S.** HPLC and TLC profiles of LF after its labelling with gallium-68, lutetium-177 and indium-111. **Figure 2S.** Total specific cell uptake after the incubation of PC3 cells with ^68^Ga-, ^177^Lu-, ^111^In-labelled LF1 within 4 or 6 h at 37 °C. Total specific cell uptake calculated as cell surface bound and internalized fraction. Nonspecific binding was determined in the presence of 1 μM Tyr^4^-BN.

## Data Availability

All data generated or analyzed during this study are included in this published article.

## Abbreviations

GPCR: G protein-coupled receptors
GRPr: Gastrin Releasing Peptide receptor
PC: Prostate cancer
BCR PC: Biochemical recurrence of prostate cancer
ER: Estrogen receptors
AAZTA^5^: 6-[Bis (carboxymethyl)amino]-1,4-bis (carboyxmethyl)-6-methyl-1,4-diazepane
DOTA: 1,4,7,10-tetraazacyclododecane-1,4,7,10-tetraacetic acid
NOTA: 1,4,7-triazacyclononane-1,4,7-triacetic acid
NODAGA: 1,4,7-triazacyclononane, 1-glutaric acid-4,7 acetic acid
HBED-CC: *N,N′*-bis-[2-hydroxy-5-(carboxyethyl)benzyl]ethylenediamine-*N,N′*-diacetic acid
Pip: 4-amino-1-carboxymethyl-piperidine
TFA: Trifluoroacetic acid
TIPS: Triisopropylsilane
EtOH: Ethanol
MeOH: Methanol
Fmoc: Fluorenylmethoxycarbonyl protecting group
DPBS: Phosphate Buffered Saline
FBS: Fetal Bovine Serum
ESI-MS: Electrospray ionization mass spectrometry
NMR: Nuclear Magnetic Resonance
LC-MS: Liquidchromatography–Mass Spectrometry
ESI-MS: Electrospray Ionization - Mass Spectrometry
TLC: Thin Layer Chromatography
RP-HPLC: Reverse-Phase High-performance liquid chromatography
HATU: 1-[Bis (dimethylamino)methylene]-1H-1,2,3-triazolo[4,5-b] pyridinium 3-oxid hexafluorophosphate
HEPES: (4-(2-hydroxyethyl)-1-piperazineethanesulfonic acid)
ACN: Acetonitrile
DMSO: Dimethyl Sulfoxide
H_2_O: Water
A_m_: Molar activities
RT: Room temperature
MRI: Magnetic resonance imaging
SPECT: Photon emission computed tomography
PET: Positron emission tomography
PSMA: Prostate Specific Membrane Antigen
PI3K: Phosphoinositide 3-kinase
AKT: Protein kinase B
mTOR: Mammalian target of rapamycin
