# Supplementary material for: Effect of the versatile bifunctional chelator AAZTA5 on the radiometal labelling properties and the in vitro performance of a gastrin releasing peptide receptor antagonist
Source: EJNMMI Radiopharm Chem. 2020 Nov 30;5:29. doi: 10.1186/s41181-020-00115-8 (PMC7704979; doi:10.1186/s41181-020-00115-8)
Supplement: Supplementary file 1 — Additional file 1: Figure 1S. HPLC and TLC profiles of LF after its labelling with gallium-68, lutetium-177 and indium-111. Figure 2S. Total specific cell uptake after the incubation of PC3 cells with 68Ga-, 177Lu-, 111In-labelled LF1 within 4 or 6 h at 37 °C. Total specific cell uptake calculated as cell surface bound and internalized fraction. Nonspecific binding was determined in the presence of 1 μM Tyr4-BN. [file 41181_2020_115_MOESM1_ESM.doc]

**Supplemental data**

**Effect of the Versatile Bifunctional Chelator AAZTA5 on the Radiometal Labelling properties and the in vitro performance of a Gastrin Releasing Peptide Receptor Antagonist**

Michael Hofstetter^1^, Euy Sung Moon^2^, Fabio D'Angelo^1^, Lucien Geissbühler^1^, Ian Alberts^1^, Ali Afshar-Oromieh^1^, Frank Rösch^2^, Axel Rominger^1^, Eleni Gourni^1^

^1^Department of Nuclear Medicine, Inselspital, Bern University Hospital, Switzerland

^2^Department of Chemistry – TRIGA site, Johannes Gutenberg - University Mainz, Germany

**Reagents and Instrumentation**

The EG1, EG2 and EG4 GIP-ligands were custom-synthesized by Peptide Specialty Laboratories

**Quality control of the radiotracers**

Chemical and radiochemical purity of the tested solutions were determined using an analytical Nucleosil 100-5 C18 column applying the conditions described in the “Reagents and Instrumentation” section.

The presence of free gallium-68 and ^68^Ga-labelled colloid in the ^68^Ga-labelled LF1 preparation was quantified by radio thin layer chromatography (Radio-TLC) using Silica gel 60-plates and two different mobile phase eluents: a) 0.1 M Na-citrate (eluent 1); b) MeOH / 1 M ammonium acetate (1 / 1, v / v) (eleuent 2). Using the first Radio-TLC eluent, the radiopeptide product and ^68^Ga-labelled colloid remain immobilized at the starting point, whereas free gallium-68 move with the mobile phase. When the second eluent is used, only the labelled peptide moves with the mobile phase / solvent front.

**Figure 1S:** HPLC and TLC profiles of LF after its labelling with gallium-68, lutetium-177 and indium-111.

**Internalization Studies**

**Figure 2S:** Total specific cell uptake after the incubation of PC3 cells with ^68^Ga-, ^177^Lu-, ^111^In-labelled LF1 within 4 or 6 h at 37°C. Total specific cell uptake calculated as cell surface bound and internalized fraction. Nonspecific binding was determined in the presence of 1 μM Tyr^4^-BN.
